# Hospital Meetings

**Published:** 1901-12-14

**Authors:** 


### HOSPITAL MEETINGS.

### MIDDLESEX HOSPITAL: QUARTERLY REPORT.

The report to the Governors of the Middlesex Hospital, for the quarter ending September 30th, states that the small-pox epidemic has caused some anxiety to the Board, and that as a precautionary measure, the hospital has been temporarily closed to visitors. The erection of a hospital laundry at Hendon has been begun; and the extension to the north-east wing of the hospital itself has been completed. The enlarged "Helena" and "Seymour" wards, with a complement of 20 beds each, have been re-opened, and the new kitchen premises, which include a servants' hall, are already occupied. In connection with these alterations the sum of £4,500 has, on the architect's certificate, been paid to the contractors on account of the cost of the work, and an expenditure of upwards of £200 on furnishing has been incurred. Owing to the serious defective condition of one of the structural piers of "Bird Ward," this ward has been temporarily closed; the defect extends to the drying-room of the laundry in the basement, and while the necessary repairs are being carried out, one of the special wards is being utilised for drying purposes. The following legacies were received during the quarter:—£1,000 from Mr. T. K. Hardie; £400 from Miss Eliz. Sloane Stanley; a quarter of the residuary estate of Mr. W. R. Whiteley, bequeathed to the Cancer Fund; and one-twelfth of the residuary estate of Mr. Matthew Whiting. Lord Howard de Walden has become a subscriber of £300; a third instalment of £100 from Major-General Sir F. Greenfell, for Cancer Research; £100, Anonymous; £100 "Research"; £210 from the Mercers' Company; £100 from the Skinners' Company; and £52 10s. from the Merchant Taylor's Company. Presents of game have been received from H.M. the King. Lord Howard de Walden has offered a further sum of £1,500 towards the provision of a recreation and athletic sports ground for the students in the medical school, on the condition that the hospital is prepared to acquire a ten-acre field for the purpose. It is proposed to advance to the Amalgamated Students' Club, from the funds of the hospital, a sum not exceeding £2,000, provided the club undertake to pay interest at the rate of 3 per cent. per annum, and to collect subscriptions for the erection of a suitable pavilion on the ground. A long-felt want will thus be supplied. The erecting, at the Convalescent Home, Clacton-on-Sea, of a pavilion for the reception of four male patients at present undergoing the open-air treatment for tuberculosis is proceeding satisfactorily. During the quarter the number of in-patients treated was 1,062, with the result shown in the subjoined analysis:—

| | |
|---|---|
| Discharged relieved ... ... ... ... ... | 690 |
| „ unrelieved ... ... ... ... ... | 58 |
| Left at own request ... ... ... ... ... | 16 |
| Deaths ... ... ... ... ... ... ... | 70 |
| Remaining in the hospital on the 1st Oct. last | 228 |
| | 1,062 |

In the cancer wards 31 patients were admitted; there were 23 deaths. In the out-patient department 12,580 patients received treatment. The total number of attendances amounted to 28,742, an average of 2 3 attendances for each patient, and 316 patients seen daily.

### LONDON HOSPITAL.

The Hon. Sydney Holland, the Chairman, presided at the Quarterly Court of Governors of the London Hospital on Wednesday, 4th inst. The report of the House Committee stated that the in-patients during the quarter numbered 3,071, of whom 1,122 were discharged cured, 1,602 relieved, while 347 died. The daily average of patients in the wards was 684. The out-patients numbered 19,504, and the dental patients and minor casualties 37,038.

In his address, embodying the salient features of the Committee's report, the Chairman said: On various occasions your Committee have had to report to you the very heavy pressure there has been in the wards of the hospital, and the growing pressure from outside on our resources. This quarter we have to report to you numbers far in excess of anything ever before reached in the history of the hospital. The total number of in-patients that have been treated is 3,071, and on November 14th we actually had 760 acutely-ill patients in the wards—a greater number than has ever been in any hospital in the United Kingdom. With the wards exactly as they used to be it would have been quite impossible to arrange for the accommodation of so many cases, but at the present time we are using the new block built in Oxford Street for many of our male cases. It is a matter for great congratulation to find all anticipations in regard to this new block fully justified in practice, and the building which has now been in use since September 18th for 80 patients will well repay a visit from those governors who have not yet seen it, and they will appreciate how deeply indebted the poor of East London are to that anonymous donor who gave £32,000 to build and equip this block. It is interesting to record with reference to our in-patients that a memorandum was prepared for the London County Council as to the way in which accidents are brought to this hospital; this memorandum showed that out of 966 accidents brought here during the space of four weeks, 893 came from within one mile of the hospital, 27 came on police ambulance, 8 on other ambulances, 118 brought in cabs and other vehicles, and the remainder were either carried or walked. These fignres are interesting when compared with the figures we lately published giving an analysis of the in-patients treated in 1900. The following is the record:—

| | | | |
|---|---|---|---|
| 5,360 | patients came from | within | 1 mile. |
| 1,869 | „ | „ | 2 miles. |
| 1,132 | „ | „ | 3 miles. |
| 2,379 | „ | „ | 6 miles. |
| 489 | „ | „ | 10 miles. |
| 925 | „ | over | 10 miles away. |

Total 12,154. The figures for the various parishes were:—Whitechapel, 1,643; Stepney, 781; St. George's, 1,150; Bethnal Green, 1,340; Mile End, 703; Limehouse, 271; Poplar, 558; West Ham, 1,961; Bow and Bromley, 874; Various, 2,873.

The improvements in the hospital are making good progress, and we hope very shortly to have the whole of the theatre floor advanced so far towards completion that it may be put in use, to the immense relief of our work. The ward on the second floor, under the theatre floor, that is to say the old George and Baker ward—which will be used eventually for children's ward—is now so far completed that it is to be occupied this week by the surgical cases from Gloucester ward; Gloucester ward will then be put into the builders' hands and rearranged with Cambridge ward which has already been in their hands for some time; and the ground floor of the old east wing will be completely remodelled, modern fittings will be introduced, and the ventilation, and lighting vastly improved; further, it has been decided to cover the whole of the walls with opalite at a cost of £750. The task of dealing with the centre block has been a most difficult and tedious one, but we hope within a few weeks now to have this in good working order and to have the whole of the receiving room open for use. The new convalescent home at Felixstowe, built out of money left by Baroness de Stern in memory of her husband, which will be known as the "Herman de Stern Convalescent Home," is nearing completion, and will be in occupation quite early in the new year. It is a charming building and will answer very fully a long-felt want in providing for convalescent patients who cannot be accepted in ordinary convalescent homes. We have, however, only 26 beds there and no provision yet for women needing convalescent help. The electric light is being rapidly installed in the hospital and nearly the whole of the centre block is now wired; orders have been given for wiring the east wing and Alexandra wing. On September 23rd Dr. Woakes tendered his resignation as senior aural surgeon. The committee recommend that Dr. H. L. Lack, M.D., F.R.C.S.Eng., should be appointed aural surgeon; but one of the candidates, Mr. Hunter Tod M.B., F.R.C.S.Eng., was felt to be so strong a candidate that, as he is an old London Hospital man, it was determined to secure his services by recommending that he should be appointed assistant aural surgeon until the termination of Mr. Mark Hovell's appointment as aural surgeon on December 1st, 1905. The committee have been considering very carefully, in conjunction with the representatives of the medical staff, the question of the dieting of patients, and a most complete diet table has now been drawn up which will ensure not only the best possible nourishment for the patients themselves, but also a complete check on any possible extravagance.

The heavy expenditure which has been incurred to meet the cost of the building is telling severely now on our funds. In order to pay off the amount which had been borrowed from our bankers and to meet current expenses it has been unanimously agreed by the committee that the stocks to the amount of £30,000 should be sold, and the approval of the governors to the sale of these stocks by the trustees is now asked. The new London Hospital Pharmacopœia which has engaged the attention of a sub-committee of the staff has now been published and found most satisfactory by all who have to use it. A unanimous vote was agreed to by the committee on October 11 cordially congratulating the Right Hon. Sir Joseph Dimsdale on his election as Lord Mayor. Sir Joseph was for very many years connected with this hospital as a member of the house committee, and his interest, we are glad to say, remains unabated. The committee recommend that the firm of Hanbury, Whiting and Co. be elected as legal advisers.

Miss Morgan, who has been matron's assistant here and sister in the hospital for many years, has been appointed matron of Addenbrooke's Hospital, Cambridge, and carries with her the good wishes of your committee for her success. Miss Sargeant has been appointed to succeed her as matron's senior assistant. Miss Macintosh has also been elected matron's assistant, and Miss Jacka has been appointed office assistant.

On the estate it is a matter of congratulation that the justices have agreed to the closing of Romford Street; the rearrangement and reorganisation of this property, which has long been desired, will be proceeded with at once.

In conclusion, the Chairman desired publicly to acknowledge the gratitude of the Committee to the Board of Guardians of West Ham who, as soon as they learned that 1,961 people from their district had used the hospital as in-patients, voted a donation of 50 guineas. Such action had come as a distinct encouragement in the quarter of the hardest work they had ever had.

The report was agreed to; the various officers were re-appointed for the ensuing 12 months, and the proceedings terminated with a vote of thanks to the Chairman.

### SURGICAL AID SOCIETY.

The Lord Mayor (Sir Joseph Dimsdale, M.P.) presided at the thirty-ninth annual meeting of the Surgical Aid Society, held on Monday, 9th instant, at the Cannon Street Hotel.

The Secretary, Mr. Tresidder, read the report for the past year. This chronicled a year of prosperity and progress which witnessed an encouraging growth both in the income of the society and in the amount of relief afforded. The committee referred in suitable terms to the death of Queen Victoria, and announced that the King had consented to become a patron of the society. The net income for the year was £18,236, an increase of £2,627—the item of annual subscriptions showing an advance of £472. The ordinary expenditure was £15,374, and of this £12,665 was paid for surgical appliances and surgeons' fees, giving a percentage of 82·5 for relief and 17·4 for management expenses. Legacies amounted to £1,500, and substantial help was again received from the City Companies, employers of labour, and others. No less than 18,282 patients were relieved, as compared with 16,714 in the preceding 12 months. This brought the weekly average up to 352, and the grand total for the 39 years to 244,486. The number of appliances supplied during the year was 27,887. In addition to the supply of these appliances, beds, carriages, batteries, etc., to the number of 346 were lent to patients suffering from temporary afflictions. After careful consideration the committee had decided for the present to apply the income from the Mackeson Fund to the relief of specially necessitous and deserving patients by liberal grants towards the cost of their appliances, and in this way 290 cases had been dealt with during the year. The confidence expressed in the last report that the declension in the contributions from the local branches was but temporary proved to be well founded; these branches had remitted £1,728 during the twelve months, an increase of nearly £200. Two changes in the personnel of the Committee of Management had to be recorded. Mr. Joseph Johnson, a highly esteemed member since 1877, died in April last; and Mr. Harry J. Powell, after 18 months' service, resigned in June. Their places had been filled by the election of Mr. G. H. Burnett and the Rev. R. Cobden Earle.

The Lord Mayor moved the adoption of the report, and congratulated the society on being one of the few charitable institutions which had not found a diminution and depletion of its resources in the last few years. The motion was seconded by the Venerable Archdeacon Sinclair and agreed to.

Mr. Alderman and Sheriff Bell moved a vote of thanks to the Treasurer, and a request to him to continue in that office. This was seconded by the Rev. N. R. Fitzpatric, who spoke of the regret felt that Mr. Grey was not able to be present, referred to the respect with which he was regarded, congratulated him on the report presented, and on his record of 40 years' service to the society, and expressed the hope that he would be richly rewarded for his efforts. The resolution was carried, as was one by Sir Wm. Treloar of thanks to the Committee of Management, and one by Mr. Sheriff Brooks Marshall of thanks to the staff. The Secretary announced gifts of £400 in connection with the meeting, and the proceedings terminated with a vote of thanks to the Chairman.

